# Radical‐Scavenging Violet Phosphorus Nanosheets for Attenuating Hyperinflammation and Promoting Infected Wound Healing

**DOI:** 10.1002/advs.202407545

**Published:** 2024-10-21

**Authors:** Zhuo Dai, Qiang Li, Meng Dang, Xiaoye Li, Ao He, Weijun Xiu, Minjin Wang, Yu Zhang, Meng Ding, Heng Dong, Yongbin Mou

**Affiliations:** ^1^ Nanjing Stomatological Hospital, Affiliated Hospital of Medical School Institute of Stomatology Nanjing University 30 Zhongyang Road Nanjing Jiangsu 210008 China; ^2^ Institute for Health Innovation and Technology Biomedical Engineering Department National University of Singapore 21 Lower Kent Ridge Road Singapore 119276 Singapore

**Keywords:** antibacterial, antioxidant, macrophage polarization, violet phosphorus nanosheets, wound healing

## Abstract

Antibacterial therapy targeting the regulation of macrophage polarization may be a useful approach for normalizing the immune environment and accelerating wound healing. Inspired by black phosphorus‐based nanoplatforms, more stable yet less‐explored violet phosphorus nanosheets (VPNSs) are expected to provide a superior solution for effectively combating bacterial infections. In this study, an average thickness of 5–7 nm VPNSs are fabricated through the liquid‐phase exfoliation method to serve as an immunoregulatory dressing for the treatment of infected wounds. VPNSs attenuated excessive reactive oxygen species (ROS) and reduced the accumulation of proinflammatory M1 macrophages, showing notable antioxidant and anti‐inflammatory properties. Comprehensive RNA sequencing further elucidated the potential immunoregulatory mechanisms of VPNSs, including modulation of the inflammatory response and enzyme regulator activity. Additionally, the inherent photothermal properties of the VPNSs contributed significantly to their antibacterial efficacy. When combined with near‐infrared laser irradiation, VPNSs showed remarkable effectiveness in reducing infection‐related complications and expediting wound healing in infected skin wound models. The rapid promotion of wound healing through ROS clearance, the regulation of macrophage polarization, and hyperthermia generation underscores the potential of the violet‐phosphorus‐based nanoplatforms as clinically viable agents for treating infected wounds. This study suggests that VPNSs are promising candidates for clinical anti‐infective and anti‐inflammatory applications.

## Graphical abstract

Violet phosphorus nanosheets (VPNSs), produced via liquid phase exfoliation, demonstrate potent antibacterial, anti‐inflammatory, and photothermal capabilities. These nanosheets proficiently manage local infections and inflammation and enhance wound healing through the scavenging of ROS and the modulation of macrophage polarization. Owing to their comprehensive therapeutic properties, VPNSs are highly promising for future clinical applications in anti‐biofilm strategies.

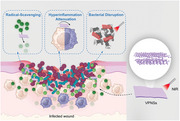

## 1 Introduction

Bacterial infection is a widespread obstacle to wound healing and skin regeneration and has emerged as a significant global health threat.^[^ ^1^ ^]^ The moist and exudative nature of wounds provides an ideal environment for bacteria to multiply.^[^ ^2^ ^]^ In response to bacterial pathogens, the immune system of healthy individuals generates antimicrobial ROS to eradicate the invading bacteria.^[^ ^3^ ^]^ While this process is generally advantageous for wound healing, uncontrolled ROS overproduction can lead to detrimental effects, such as DNA damage, protein degradation, lipid peroxidation, and excessive inflammation.^[^ ^4^ ^]^ Excessive accumulation of ROS and inflammation delay wound healing and exacerbate tissue injury.^[^ ^5^ ^]^ Consequently, effective wound healing necessitates the regulation of active oxygen levels within the body, and restoration of the equilibrium in oxidative‐reductive processes.^[^ ^6^ ^]^ Currently, the primary therapeutic agents for scavenging ROS are antioxidants, which can be categorized into three groups: small molecules, artificial nanocatalytic particles, and natural antioxidant enzymes.^[^ ^7^ ^]^ They have been extensively explored for repairing injured tissues.^[^ ^8^ ^]^ Unfortunately, owing to the complexity of the wound microenvironment, most treatments have primarily focused on scavenging ROS rather than preventing their persistent and excessive production by regulating inflammation levels, resulting in limited efficacy.^[^ ^9^ ^]^ Therefore, there is an urgent need to develop novel biomaterials with dual target pathogens and immune microenvironments to improve wound treatment.

2D nanomaterials have unique nanosheet structures and large surface areas, along with exceptional thermal, optical, and mechanical properties. These features make them highly promising for applications in drug delivery, antibacterial treatments, and tissue engineering.^[^ ^10^ ^]^ Black phosphorus (BP), an allotrope of phosphorus, exhibits good biocompatibility because it is metabolized to phosphate in living organisms.^[^ ^11^ ^]^ The layered crystal structure of BP stacked through van der Waals forces, akin to graphite, allows the formation of a single or few layers of 2D BP nanosheets (BPNSs) through a liquid‐phase exfoliation strategy owing to weak interlayer connections. BPNSs have applications in various biomedical fields, including drug delivery,^[^ ^12^ ^]^ photothermal therapy,^[^ ^13^ ^]^ antibacterial therapy,^[^ ^11^ ^]^ antitumor therapy,^[^ ^14^ ^]^ and tissue engineering.^[^ ^15^ ^]^ In addition, the zerovalent state and lamellar structure with a large surface area endow BPNSs with excellent ROS‐scavenging capabilities, enhancing their potential to treat inflammation‐related diseases.^[^ ^16^ ^]^ However, BPNSs are easily oxidized by oxygen in aqueous solutions, degrade quickly, and have poor stability, limiting their wide application in the biomedical field. Violet phosphorus is an allotrope of biological phosphorus.VPNSs offer exceptional thermal stability and chemical resistance compared with BPNSs, making them particularly advantageous for biomedical applications that require long‐term exposure to physiological conditions. Additionally, their unique 2D structure increases their surface area, providing more sites for interaction with biological entities, which is crucial for targeted drug delivery and photothermal therapy. The thermal decomposition temperature (52 °C) of VPNSs is higher than that of black phosphorus, suggesting that violet phosphorus is a more stable allotrope and benefits from photothermal treatment. VPNSs can be obtained through both mechanical and solution exfoliation methods under ambient conditions, while black phosphorus must be obtained under protective conditions.^[^ ^17^ ^]^ Thus, the use of VPNSs has been studied in the field of tumor treatment.^[^ ^18^ ^]^ In addition, the outer layer of VPNSs is coated with numerous sub‐nanometer needles, possessing a high surface energy that enhances their interaction with the bacterial cell membrane, resulting in physical damage and ultimately leading to bacterial death.^[^ ^19^ ^]^ Their excellent properties endow them with significant potential in the field of antibacterial treatments.

**Scheme 1:**
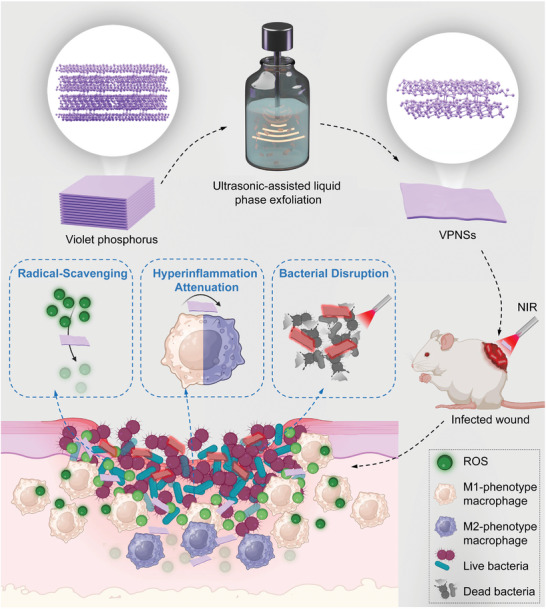
Schematic illustration of the formation of VPNSs and the infected wound management via VPNSs. VPNSs, obtained via liquid‐phase exfoliation, have emerged as an effective immunoregulatory dressing for treating infected wounds by attenuating ROS and reducing M1 phenotype macrophage accumulation, thus displaying antioxidant and anti‐inflammatory properties, while their photothermal properties enhance their antibacterial capabilities. Demonstrating significant efficacy under NIR irradiation, VPNSs facilitate rapid infection wound healing through ROS clearance, regulation of macrophage polarization regulation, and hyperthermia generation.

In this study, we introduced a novel strategy for treating infected wounds using VPNSs, as illustrated in **Scheme** **1**. VPNSs are employed as multifunctional therapeutic agents to leverage the synergistic effects of ROS clearance, macrophage polarization regulation, and hyperthermia generation. The ability of VPNSs to clear ROS plays a vital role in the modulation of inflammatory responses, reducing oxidative stress at wound sites and preventing tissue damage. Additionally, VPNSs facilitate the shift of macrophage polarization from a pro‐inflammatory M1 phenotype to a reparative M2 phenotype, enhancing inflammation resolution and promoting tissue repair and regeneration. The generation of hyperthermia upon exposure to near‐infrared (NIR) light not only directly restricts bacterial proliferation by raising local temperatures but also improves the overall therapeutic efficacy of VPNSs. These mechanisms collectively exert robust antibacterial and anti‐inflammatory effects. The dual antimicrobial and anti‐inflammatory properties of VPNSs, combined with their ability to modulate immune responses, establish VPNSs as attractive agents for addressing complex wound‐care challenges, especially in chronic wounds and those resistant to conventional treatments.

## 2 Results and Discussion

### 2.1 Characterization of VPNSs

**Figure 1:**
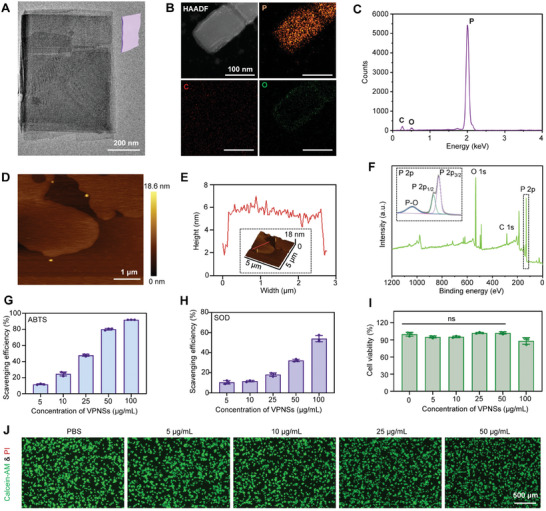
Characterization of VPNSs. A) High‐resolution transmission electron microscopy (HR‐TEM) image of a representative VPNS. B) High‐angle annular dark‐field (HAADF) and elemental mapping images display phosphorus (P), carbon (C), and oxygen (O) distribution in VPNSs. C) EDS analysis of VPNSs. D) Atomic force microscopy (AFM) images of a representative VPNSs sample. E) Height profiles and thickness measurements of a representative VPNSs sample. F) X‐ray photoelectron spectroscopy (XPS) spectra of VPNSs. G) ABTS assay showing the scavenging activity of VPNSs toward free radicals. H) Superoxide dismutase (SOD) activity analysis of VPNSs. I) Evaluation of VPNSs biocompatibility at indicated concentrations using the CCK‐8 assay. J) Confocal laser scanning microscopy (CLSM) images of RAW264.7 cells cultured with varying concentrations of VPNSs. Data are presented as mean ± standard deviation (SD) (*n* = 3). ns indicates not significant.

The VPNSs were synthesized using a liquid‐phase exfoliation strategy. The TEM images revealed the nanosheet morphology of the VPNSs (**Figure** **1**A; Figure S1, Supporting Information). The VPNSs were composed of [P2][P8][P2][P9] tubular strands units, which were arranged parallel in‐plane and covalently linked vertically through [P9] to form a layered structure,^[^ ^17^ ^]^ as shown in Figure S2A (Supporting Information). These layers are stacked along the *c*‐axis via van der Waals forces, ultimately forming violet phosphorus. The asymmetric unit of violet phosphorus drawing by Oak Ridge thermal ellipsoid plot (ORTEP) is shown in Figure S2B (Supporting Information). Energy‐dispersive X‐ray spectroscopy (EDS) elemental mapping confirms that the prepared VPNSs predominately consisted of phosphorus with only trace amounts of oxygen (Figure 1B). The quantitative results also show minimal levels of oxygen and carbon (Figure 1C), demonstrating that the obtained VPNSs were relatively stable and no serious oxidation occurred. Atomic force microscopy (AFM) images reveal the layered structure and flat surface of the VPNSs (Figure 1D). The high‐resolution AFM measurements show that the VPNSs had an average thickness of 5–7 nm (Figure 1E). Wide‐scan X‐ray photoelectron spectroscopy (XPS) analysis shows P 2p, O 1s, and C 1s peaks, revealing the presence of these elements in the VPNSs. The high‐resolution XPS spectrum displays three peaks corresponding to P 2p_3/2_ and phosphate P 2p_1/2_ (Figure 1F). The O 1s XPS spectrum shows only one photoelectron peak with a binding energy of ≈532 eV, indicating that only surface‐adsorbed oxygen species and not lattice oxygen are present in the violet phosphorus sample^[^ ^20^ ^]^ (Figure S3, Supporting Information). This result proves that VPNSs have excellent antioxidant properties. Violet phosphorus is a layered phosphorus allotrope, more stable than black phosphorus.^[^ ^21^ ^]^ The electron diffraction patterns and zeta potentials of the VPNSs are shown in Figures S4 and S5 (Supporting Information). Previous studies on BPNSs have highlighted their unique few‐layer structure, which is stacked via weak Van der Waals forces and is characterized by the zerovalent state of phosphorus, facilitating rapid electron transfer.^[^ ^16^ ^]^ This property is particularly crucial for oxidative reactions.

Reactive oxygen and nitrogen species (ROS/RNS) play crucial roles in the pathogenesis of wound healing, closely associated with cellular oxidative stress. To assess the antioxidant capabilities of VPNSs, evaluations were conducted using the 2,2'‐azino‐bis (3‐ethylbenzthiazoline‐6‐sulfonic acid) (ABTS) radical inhibition assay and measurements of superoxide dismutase (SOD)‐like activity. The ABTS assay was employed to quantify the total antioxidant capacity of the VPNSs, reflecting their efficacy in neutralizing free radicals. The results indicated that VPNSs, at a concentration of 100 µg mL^−1^, were capable of scavenging ROS with an impressive efficiency of 90% (Figure 1G). Additionally, as a critical antioxidant enzyme, SOD acted as an endogenous scavenger of superoxide anions and protected cells against oxidative stress.^[^ ^22^ ^]^ The robust ROS‐scavenging capability of the VPNSs was confirmed by SOD assay (Figure 1H). Biosafety, a fundamental requirement for biomaterial applications, was evaluated in macrophages and keratinocytes, which are the two primary cell types involved in wound healing. The CCK‐8 assay results show limited cytotoxicity of VPNSs on RAW264.7 cells even at 100 µg mL^−1^ (Figure 1I). The cytotoxicity in RAW264.7 and HaCaT cells was further assessed in vitro through apoptosis analysis using calcein‐AM/propidium iodide (PI) and Annexin V/PI staining assays. The calcein‐AM/PI assay indicated that the cell viability in both cell lines remained above 95% in the VPNSs group, suggesting excellent biocompatibility (Figures 1J; Figure S6, Supporting Information). Furthermore, apoptosis rates were generally low (<5%) across different concentrations in both RAW264.7 and HaCaT cells, demonstrating the safety of VPNSs for treatment (Figures S7 and S8, Supporting Information). In summary, VPNSs exhibited superior biocompatibility and antioxidative activities, underscoring their potential benefits in wound healing treatments.

### 2.2 Cellular Uptake and In Vitro Anti‐Inflammatory Effect of VPNSs

**Figure 2:**
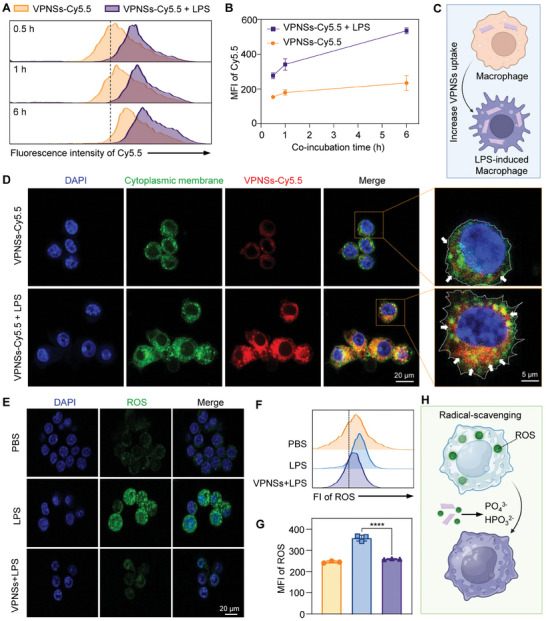
In vitro radical‐scavenging properties of VPNSs. A) Representative flow cytometry (FCM) histograms and B) mean fluorescence intensity (MFI) of time‐dependent cellular uptake in PBS‐ or LPS‐stimulated RAW264.7 cells. C) Schematic illustration of increased VPNSs uptake by LPS‐stimulated macrophages. D) CLSM observations of VPNSs cellular uptake, with and without LPS stimulation. E) CLSM images showing ROS green fluorescent expression in RAW264.7 cells. Blue: DAPI‐stained nuclei; green: ROS. F) Representative FCM histograms and G) MFI of ROS production in LPS‐stimulated RAW264.7 cells, with and without VPNSs treatment. H) Schematic illustration of VPNSs functioning as radical‐scavenging agents. Data are shown as mean ± SD (*n* = 3). The statistical significance of differences between the indicated groups was analyzed using one‐way ANOVA. ****p < 0.0001.

Cellular uptake of nanomaterials is a pivotal process that triggers biological responses within cells. We explored the uptake of VPNSs by both LPS‐stimulated and unstimulated macrophages. Notably, the phagocytosis of VPNSs by LPS‐induced polarized macrophages was significantly greater compared to that in nonpolarized macrophages (**Figure** **2**A–C). Confocal laser scanning microscopy (CLSM) confirmed that the VPNSs were effectively engulfed by the macrophages while maintaining their cell structures and not inducing cytotoxic effects(Figure 2D). Flow cytometry was used to assess the protective effects of VPNSs against H₂O₂‐induced apoptosis in macrophages. Quantitative analysis showed that the apoptotic rate in the H₂O₂ group was significantly elevated at 23.93 ± 1.50%, compared to the control group. With VPNSs treatment, the apoptotic rate decreased to 9.25 ± 0.53%, indicating that VPNSs could mitigate H₂O₂‐mediated cellular damage and reduce apoptosis (Figure S9, Supporting Information). To evaluate the ROS scavenging ability of the VPNSs during the LPS‐triggered inflammatory response, RAW264.7 macrophages were exposed to LPS (200 ng mL^−1^) for 12 h. The production of ROS was monitored using CLSM, where the LPS group exhibited a significant increase in green fluorescence intensity, indicating enhanced ROS production compared to control groups (Figure 2E). The lower fluorescence intensity of VPNSs‐treated cells showed that VPNSs could reduce LPS‐induced ROS production in macrophages. The mean fluorescence intensity (MFI) of ROS was quantitatively analyzed by flow cytometry, and the results are shown in Figure 2F,G. These measurements confirmed that LPS significantly increased intracellular ROS levels, whereas treatment with VPNSs considerably reduced ROS fluorescence intensity, indicating the effective mitigation of LPS‐triggered ROS production by VPNSs. These findings demonstrate that VPNSs possess potent anti‐inflammatory properties by inhibiting ROS production in macrophages, highlighting their potential as therapeutic agents for inflammatory conditions (Figure 2H).

### 2.3 Transcriptomic Analysis by RNA‐Sequencing

**Figure 3:**
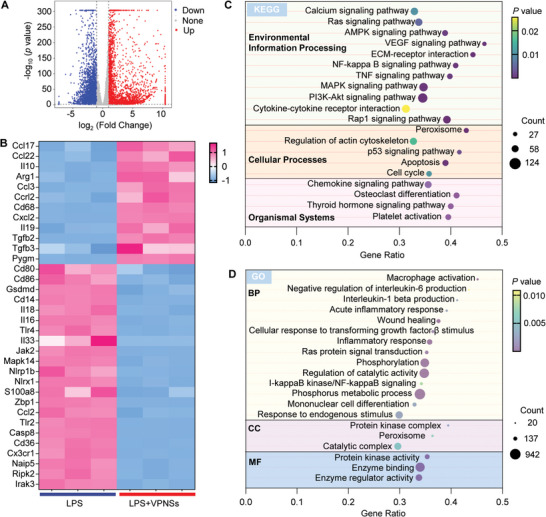
Key Differentially Expressed Genes in LPS‐Stimulated Macrophages Regulated by VPNSs. A) Volcano plot detailing the differentially expressed genes (DEGs) between LPS and LPS+VPNSs‐treated RAW264.7 cells. Red indicates up‐regulated DEGs; blue denotes down‐regulated DEGs; grey represents non‐significant DEGs. B) Heatmap illustrating the expression profiles related to inflammation‐associated DEGs in RAW264.7 cells treated with LPS or LPS+VPNSs. C) Kyoto Encyclopedia of Genes and Genomes (KEGG) and D) Gene Ontology (GO) enrichment analyses of the DEGs between LPS‐ and LPS+VPNSs‐treated RAW264.7 cells. The Figures highlight the top 20 enriched terms. Dot size corresponds to the number of DEGs associated with each term, and color reflects the level of statistical significance. BP represents biological processes; CC stands for cellular components; MF denotes molecular functions.

Bacterial infections can impede wound repair, leading to persistent inflammation within the wound microenvironment, which is a significant hurdle in chronic wounds.^[^ ^23^ ^]^ Macrophages play a pivotal role in healing by regulating the immune response at the wound site. Following the recruitment of monocytes to an inflamed wound, macrophages undergo polarization into M1 proinflammatory or M2 anti‐inflammatory phenotypes, which significantly influence the wound healing process.^[^ ^24^ ^]^ Although an appropriate inflammatory response aids early wound repair, excessive inflammation, primarily due to disrupted polarization from M1 to M2, results in the predominant accumulation of M1 macrophages.^[^ ^4a^ ^]^ M1 macrophages overproduce proinflammatory cytokines and various ROS, leading to chronic nonhealing infected wounds. Preliminary evidence suggests that VPNSs possess nanoenzymatic activity capable of scavenging multiple free radicals and modulating immune responses. To further elucidate this biological mechanism, RNA sequencing was performed on macrophages treated with LPS or LPS + VPNSs to compare transcript levels. The transcriptomes of LPS + VPNSs‐treated RAW264.7 cells displayed distinctive characteristics compared to those treated with LPS alone (Figure S10, Supporting Information). The analysis identified 5802 differentially expressed genes (DEGs), with 3151 upregulated and 2651 downregulated (Figure S11, Supporting Information). The heatmap illustrated significant variability in the interaction densities and clustering between the two treatment groups (Figures S12 and S13, Supporting Information). Clustering volcano plots and heatmaps of differential gene expression indicated that downregulated genes, such as CD80 and CD86, were associated with the M1 phenotype of macrophages (**Figure** **3**A,B). Further analysis using the Kyoto Encyclopedia of Genes and Genomes (KEGG) data revealed that the top 20 enriched pathways, including the TNF and MAPK signaling pathways, were predominantly related to immunoregulation (Figure 3C). Additionally, the enriched Gene Ontology (GO) terms covered various aspects of the inflammatory response, enzyme regulator activity, phosphorus metabolic processes, cytokine activity, and biological regulation (Figure 3D). Transcriptomic analysis demonstrated that most DEGs in VPNSs‐treated LPS‐induced macrophages were closely associated with anti‐inflammatory and antioxidative stress responses. To investigate the network connections among DEGs, the protein–protein interaction (PPI) network was also visualized, which showed that the expression of CD80 and CD86 genes was downregulated and highly correlated in the connection network (Figure S14, Supporting Information).

**Figure 4:**
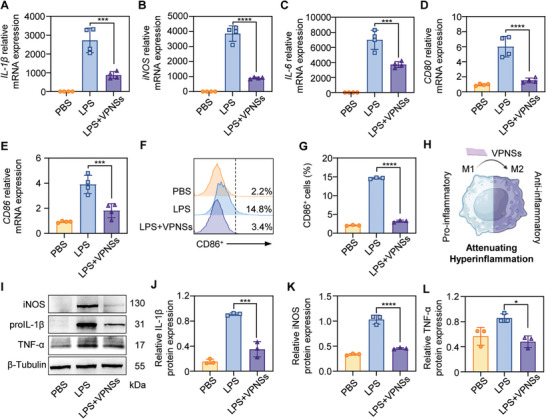
Anti‐inflammatory effect of VPNSs. Expression of A) *IL‐1β*, B) *iNOS*, C) *IL‐6*, D) *CD80*, and E) *CD86* mRNA in RAW264.7, as determined by RT‐qPCR. Data are presented as mean ± SD (*n* = 4), ***p < 0.001; ****p < 0.0001. F) Representative FCM histograms and G) proportions of CD86^+^ cells in RAW264.7 stimulated by LPS with or without VPNSs. H) Schematic illustration of VPNSs regulating macrophage polarization. I) Western bolt analysis of the protein levels of inflammatory factors in RAW264.7 cells. Quantitative analysis of J) IL‐1β, K) iNOS, and L) TNF‐α protein expression. Data are presented as mean ± SD (*n* = 3) *p < 0.05; ***p < 0.001; ****p < 0.0001.

VPNSs possess strong antioxidant properties, and RNA‐seq results indicate a strong correlation between inflammatory pathways and enzyme activity. Therefore, we investigated the role of VPNSs in inhibiting macrophage M1 polarization through their antioxidant and anti‐inflammatory effects in vitro. Considering that wound healing is closely associated with the M1 polarization of macrophages, the antioxidative and anti‐inflammatory effects of VPNSs on M1 macrophages were further studied. To further investigate the anti‐inflammatory effect of VPNSs, the mRNA expression levels of inflammatory biomarkers were evaluated using RT‐qPCR. With the treatment of VPNSs, the expression levels of proinflammatory cytokines were significantly downregulated compared with the LPS‐treated group, including *IL‐1β*, *iNOS*, *IL‐6*, *CD80*, and *CD86*, marking the polarization of macrophages into the M1 phenotype in gene level (**Figure** **4**A–E). Flow cytometry revealed that LPS stimulation markedly increased CD86 expression in macrophages, whereas VPNSs significantly reduced CD86 expression (Figure 4F,G; Figure S15, Supporting Information), the formation of pseudopodia in LPS‐treated macrophages, and the increase in proinflammatory cytokines (Figure 4H). CLSM results showed that LPS‐stimulated macrophages expressed high levels of iNOS accompanied by the growth of pseudopodia, whereas VPNSs treatment can reduce the expression of iNOS in cells accompanied by a reduction in pseudopodia growth (Figure S16, Supporting Information). A robust anti‐inflammatory effect could be induced by VPNSs via reducing the protein expression of inflammatory factors (IL‐1β, iNOS, TNF‐ɑ; Figure 4I–K). Their uniform downregulation suggests that VPNSs oppose LPS‐induced polarization of macrophages into classically activated M1 macrophages.

Collectively, these in vitro findings suggest that VPNSs exhibit anti‐inflammatory effects by directly scavenging excessive ROS in inflammatory macrophages, regulating subsequent signaling pathways, and inhibiting the secretion of proinflammatory molecules.

### 2.4 In Vitro Photothermal Antibacterial Properties of VPNSs

**Figure 5:**
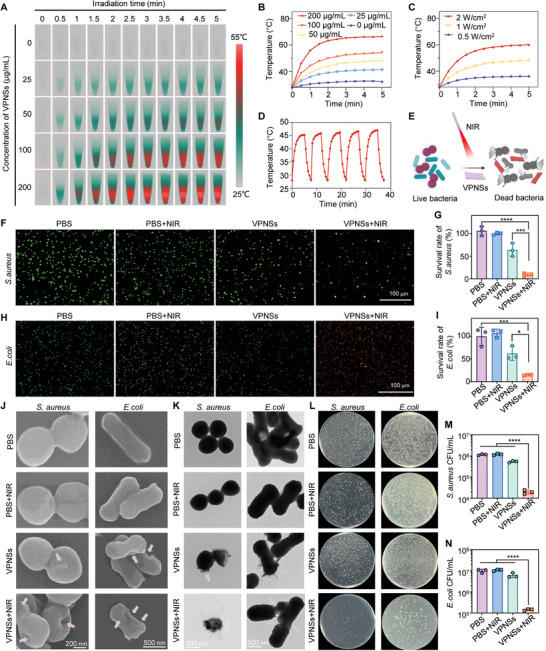
In vitro photothermal antibacterial properties of VPNSs. A) Thermal infrared images and B) photothermal response curves of water and VPNSs suspensions at concentrations of 25, 50, 100, and 200 µg mL^−1^ under 808 nm NIR laser irradiation at a power density of 1.0 W cm^−^^2^. C) Temperature profiles of a 50 µg mL^−1^ VPNSs solution under varying laser power densities of 0.5, 1.0, and 2.0 W cm^−^^2^. D) Photothermal stability curves of a 50 µg mL^−1^ VPNSs solution demonstrated through 808 nm laser on/off cycles at a power density of 1.0 W cm^−^^2^. E) Schematic illustration depicting VPNSs functioning as photothermal antibacterial agents. F,G) CLSM images and quantitative analysis of bacterial survival rates for *S. aureus*. H,I) CLSM images and quantitative analysis of bacterial survival rates for *E. coli* after treatments with PBS, PBS + NIR, VPNSs, and VPNSs + NIR. J) SEM and K) HRTEM images of *S. aureus* and *E. coli* post‐treatment with PBS, PBS + NIR, VPNSs, and VPNSs + NIR. L) Photographs of bacterial broth plates of *S. aureus* and *E. coli* and colony‐forming units (CFUs) count of M) *S. aureus* and N) *E. coli* post‐treatment with PBS, PBS + NIR, VPNSs, and VPNSs + NIR. Data are presented as mean ± SD (*n* = 3). Significance levels are indicated: ***p < 0.001; ****p < 0.0001.

To assess the photothermal capability of the VPNSs, we measured the temperature increments under 808 nm NIR laser irradiation. After 5 min of exposure at a power density of 1.0 W cm^−^^2^, the temperature of VPNSs at concentrations of 25, 50, 100, and 200 µg mL^−1^ reached 41.5, 48.3, 54.3 and 66.4 °C, respectively (**Figure** **5**A,B). For comparison, the temperature of irradiated pure water under identical conditions rose only to 32.3 °C. Additionally, we recorded the temperature changes under varying power densities of 808 nm NIR irradiation. The results indicated that an increase in the power density correspondingly increased the maximum temperature achievable by the VPNSs (Figure 5C), confirming that both the concentration and power density are critical factors influencing the photothermal effect. To assess the NIR photostability of VPNSs, we performed five on/off cycles of NIR laser irradiation (Figure 5D; Figure S17, Supporting Information). The temperature rose by 17 °C during the first cycle, with no significant decrease observed after five cycles, demonstrating the photostability of VPNSs. The photothermal conversion efficiency (𝜂) of VPNSs could reach 30.97%, suggesting great photothermal conversion efficiency (Figure S18, Supporting Information).

To evaluate the photothermal antibacterial efficacy of the VPNSs, we selected typical infectious pathogens, *Staphylococcus aureus* (*S. aureus*) and *Escherichia coli* (*E. coli*), representing Gram‐positive and Gram‐negative bacteria, respectively (Figure 5E). To balance between avoiding tissue overheating and achieving an effective photothermal response, we used a VPNSs concentration of 50 µg mL^−1^ and a power density of 1.0 W cm^−^^2^ for subsequent experiments. The antimicrobial efficacy of VPNSs under 808 nm NIR laser irradiation was elucidated using confocal laser scanning microscopy. A live/dead bacterial viability assay employing green SYTO 9 dye, which permeates both intact and compromised cells and red PI dye, which only enters cells with compromised membranes, was used to demonstrate the antibacterial activity.^[^ ^25^ ^]^ The relative antibacterial efficiencies against *S. aureus* (Figure 5F,G) and *E. coli* (Figure 5H,I) were 90.45 ± 3.22% and 85.13 ± 0.89% for the VPNSs + NIR group, respectively. Morphological changes in the bacteria were examined using Scanning electron microscopy (SEM) and high resolution transmission electron microscopy (HR‐TEM). *E. coli*, typically rod‐shaped, and *S. aureus*, generally spherical, exhibited slight post‐treatment cell membrane shrinkage. Following NIR irradiation, significant membrane destruction was observed in bacteria treated with VPNSs + NIR (Figure 5J,K). Additionally, bacterial cultures exposed to VPNSs under NIR irradiation formed substantially fewer colonies on agar plates, as shown in Figure 5L–N. The survival rate of bacteria subjected to the combined VPNSs and NIR treatment was markedly improved compared to that of those treated with either photothermal action or VPNSs alone, highlighting a synergistic antibacterial effect.

### 2.5 VPNSs‐Mediated In Vivo Infected Wound Photothermal Treatment

**Figure 6:**
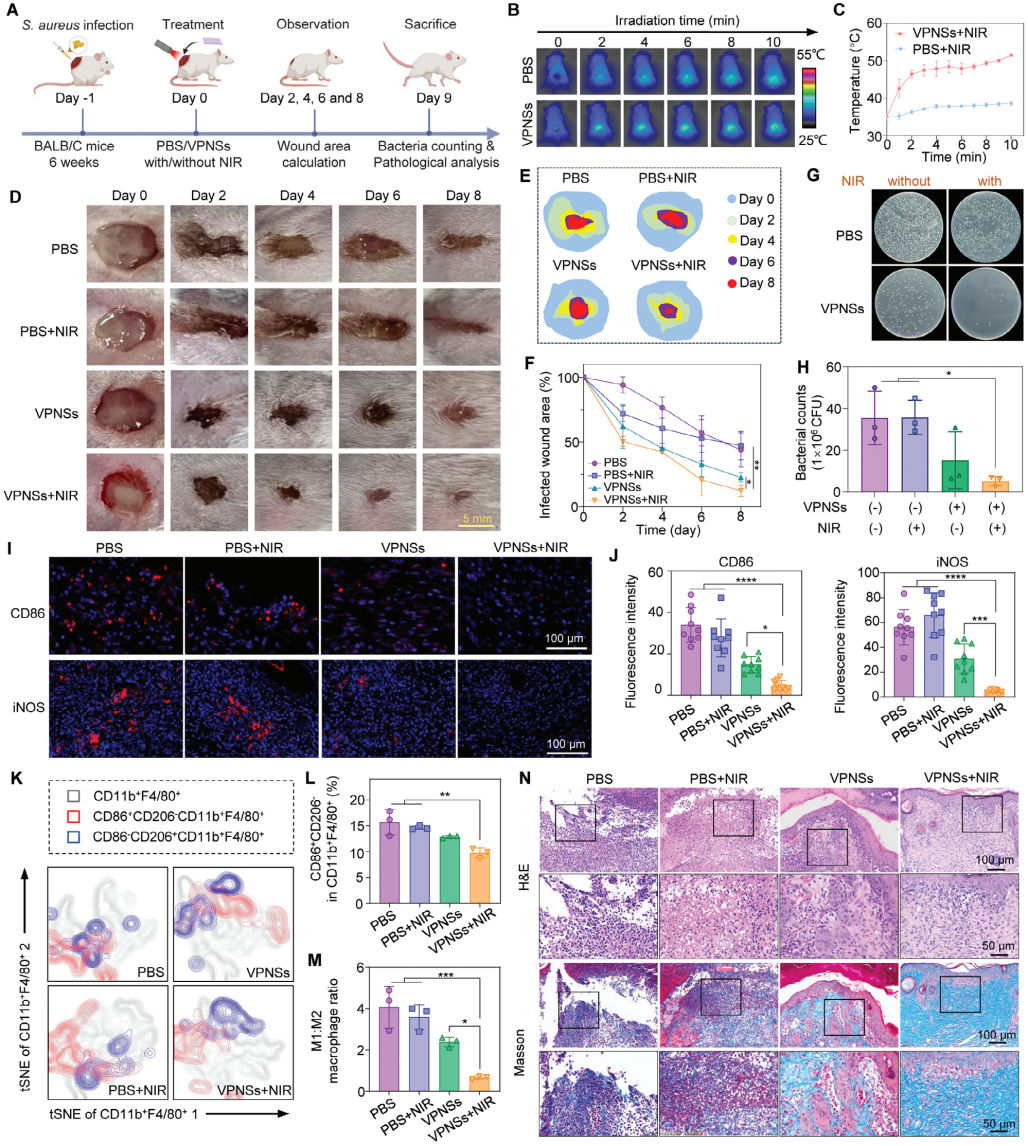
In vivo treatment of *S. aureus* infected wounds. A) Schematic illustration of the *S. aureus* biofilm‐infected mouse model and experimental protocol. B) Thermal images of mice treated with VPNSs + NIR and PBS + NIR. C) Quantification of the temperature increases in these groups. D) Representative photographs and E) schematic illustrations of *S. aureus*‐infected wounds under various treatments on days 0, 2, 4, 6, and 8. F) Quantitative analysis of wound areas in mice treated with PBS, PBS + NIR, VPNSs, or VPNSs + NIR on days 0, 2, 4, 6, and 8 (*n* = 3). G) Photographs of *S. aureus* bacterial colonies and H) viability analysis of *S. aureus* from tissue homogenates post‐treatment across different groups on day 9 (*n* = 3). I) Immunofluorescent staining for CD86 and iNOS across various treatment groups. J) Semiquantitative analysis of CD86 and iNOS expression from immunofluorescent images (*n* = 9). K) FCM analysis of CD11b^+^F4/80^+^CD86^+^CD206^−^ and CD11b^+^F4/80^+^CD86^−^CD206^+^ macrophage clusters in different treatments on day 9. Quantification of L) CD11b^+^F4/80^+^CD86^+^CD206^−^ macrophages and M) M1:M2 macrophage ratio in various treatments on day 9 (*n* = 3). N) Histological analysis of skin tissues using H&E and Masson's trichrome staining on day 9, illustrating the effects of different treatments. The statistical significance of differences between the indicated groups was analyzed using one‐way ANOVA. *p < 0.05, **p < 0.01, ***p < 0.001 and ****p < 0.0001.

Encouraged by the favorable outcomes observed in vitro, we used an *S. aureus* skin infection mouse model, representing a pre‐clinically relevant scenario for wound healing (**Figure** **6**A). We conducted a full‐thickness skin incision and *S. aureus* infection on the backs of the mice, following which various treatments were applied. The temperature of the infected wound site dressed with VPNSs reached ≈51 °C under NIR irradiation, which was significantly higher than the minimal increase observed in the PBS group (Figure 6B,C). This increase suggests efficient photothermal conversion by the VPNSs, which is critical for achieving the desired therapeutic outcomes. First, comprehensive in vivo toxicity studies are imperative for evaluating the long‐term safety of topical applications involving VPNSs and VPNSs + NIR treatments. Our observations indicated no adverse effects on the major organs of the mice after treatment, suggesting good biocompatibility of the VPNSs (Figure S19, Supporting Information). In addition, the levels of ROS in the VPNSs‐treated groups with or without NIR were significantly lower than that of the PBS group in infected skin wounds on the 3th day post‐treatment (Figure S20, Supporting Information). Next, as shown in Figure 6D,E, wounds in the VPNSs + NIR group exhibited superior healing outcomes compared to the other groups. By the 8th day, the wound area in the VPNSs + NIR‐treated mice was significantly reduced by 87.7%, whereas only 52% recovery was observed in the PBS group (Figure 6F). Additionally, a significant reduction in bacterial colonies was observed in the VPNSs + NIR group by the 9th day (Figure 6G,H). These results not only demonstrate the potent antibacterial capability of VPNSs in vivo but also highlight their potential as effective photothermal agents for clinical wound management.

Immunofluorescence staining was employed on the 9th day post‐treatment to analyze the distribution of M1 macrophages in the skin tissue sections, as shown in Figure 6I. Staining highlighted the pronounced presence of CD86‐labeled cells in both the PBS and PBS + NIR groups. By contrast, the VPNSs group showed a reduced number of inflammatory cells, with the VPNSs + NIR group displaying the fewest CD86^+^ cells. This pattern was similarly reflected by iNOS immunofluorescence staining. Statistical analysis of the mean fluorescence intensity (Figure 6J) indicated statistically significant differences between the groups. To investigate macrophage polarization from the M1 to M2 phenotype, wound tissues were processed to prepare single‐cell suspensions, and the macrophage markers CD11b and F4/80 were labeled for analysis by flow cytometry. The distributions of macrophage populations across different treatment groups are illustrated in Figure 6K. The flow cytometry results revealed that VPNSs+NIR treatment significantly reduced the proportion of CD11b^+^F4/80^+^CD86^+^CD206^−^ M1 macrophages and enhanced the proportion of CD11b^+^F4/80^+^CD86^−^CD206^+^ M2 macrophages compared to the other groups (Figure 6L; Figure S21, Supporting Information). Consequently, the M1:M2 macrophage ratio was significantly decreased (Figure 6M), highlighting the potent ability of VPNSs + NIR to suppress local inflammation and promote wound healing by modulating macrophage polarization in bacterial‐infected wounds at the late stage of infection. The secretion levels of proinflammatory cytokines (IL‐1β, TNF‐α, and IL‐6) in the VPNSs‐treated groups were lower than those in the PBS‐treated groups (Figure S22, Supporting Information). Further histological analyses were performed to evaluate the progression of wound healing. H&E and Masson's trichrome staining results, as shown in Figure 6N, revealed that the epidermal structure in the VPNSs + NIR group was more intact than that in the other groups, demonstrating increased collagen deposition and fewer inflammatory cells. Collagen deposition, a critical indicator of effective wound healing, was markedly higher in the VPNSs + NIR group, with collagen fibers forming a dense network structure, indicating superior tissue repair quality. These findings underscore the therapeutic potential of VPNSs + NIR in enhancing wound healing by efficiently shifting macrophage polarization toward a reparative phenotype and fostering an environment conducive to tissue regeneration and repair.

## 3 Conclusion

In this study, we report that VPNSs‐mediated phototherapy significantly enhances the healing of bacterial‐infected wounds, driven by the synergistic effects of photothermal action and immunomodulation. VPNSs exhibit excellent biosafety, effective radical scavenging, and robust photothermal stability, which contribute to their therapeutic efficacy. Notably, our findings reveal that VPNSs inhibit the polarization of macrophages to the M1 phenotype, modulate inflammatory responses, and demonstrate remarkable antibacterial efficacy under NIR irradiation both in vitro and in vivo. Furthermore, VPNSs play a crucial role in immune regulation within bacterially infected full‐thickness wounds, attenuating hyperinflammation and promoting wound healing. Overall, this study highlights the exceptional antibacterial and immunomodulatory capabilities of VPNSs, positioning them as promising agents for the treatment of bacterial‐infected wounds.

## 4 Experimental Section

### Characterization of VPNSs

VPNSs were prepared using a modified sonication‐assisted liquid exfoliation method. Initially, 100 mg of bulk violet phosphorus was ground in a mortar and subsequently dispersed in 100 mL of methanol. The mixture was subjected to ultrasonication for 12 h in an ice bath using an ultrasonic homogenizer. Following ultrasonication, the dispersion was first centrifuged at 1000 rpm for 5 min to separate unexfoliated bulk material. The supernatant was then centrifuged at 13 000 rpm for 15 min to isolate ultrathin VPNSs. Finally, the VPNSs were purified by a rotary evaporator and resuspended in PBS. The morphology and composition of VPNSs were observed by high‐resolution transmission electron microscopy (HR‐TEM, Talos F200X) and high‐angle annular dark‐field scanning transmission electron microscopy. The Zeta potentials of VPNSs were measured using a Brookhaven ZetaPALS analyzer (Brookhaven Instruments, Holtsville, NY, USA). SEM measurements were performed using a Hitachi‐S4800 microscope operated at 5 kV (Tokyo, Japan). AFM imaging was performed with SPM‐9700 (Shimazu, Kyoto, Japan) AFM instrument. A PHI 5000 VersaProbe (ULVAC‐PHI, Chigasaki, Japan) was used to perform XPS of VPNSs.

### Cellular Uptake Efficiency

The RAW264.7 cell line was obtained from the Cell Bank of the Chinese Academy of Sciences (Shanghai, China). RAW264.7 cells were cultured in DMEM medium supplemented with 10% FBS and 1% P/S at 37 °C under a 5% CO_2_ atmosphere. RAW264.7 cells were stimulated by LPS (200 ng mL^−1^) for 12 h and polarized into the M1 phenotype. The fluorescent dye Cy5.5 was modified to VPNSs by covalent bonding to evaluate their cellular uptake. RAW264.7 cells were cultured in 12‐well plates at a density of 2 × 10^5^ cells per well for flow cytometry analysis, and in confocal dishes at a density of 1 × 10^5^ for confocal laser scanning microscopy (CLSM) studies. Following cell adhesion, PBS or LPS (200 ng mL^−1^) was added and the cells were incubated for 12 h. Subsequently, VPNSs‐Cy5.5 (50 µg mL^−1^) was introduced to the cultures for varying durations of 0.5, 1, and 6 h, with triplicate samples at each time point. Post‐treatment, cells were washed thrice with PBS, and harvested, and the mean fluorescence intensity (MFI) of Cy5.5 was measured using flow cytometry. Simultaneously, cells treated for 6 h were prepared for confocal fluorescence imaging. These cells were stained with 1 µL of fluorescein isothiocyanate (FITC)‐labeled cytoplasmic membrane stain and incubated for 10 min at 37 °C in the dark. Following staining, cells were washed three times with PBS and fixed with 4% paraformaldehyde (PFA) for 15 min at room temperature. After another series of PBS washes, the cell nuclei were stained with a DAPI‐containing anti‐fluorescence quencher. Imaging was conducted using a Nikon A1 CLSM (Nikon; Tokyo, Japan).

### In Vitro Cytotoxicity of VPNSs

The HaCaT cell line was also obtained from the Cell Bank of the Chinese Academy of Sciences (Shanghai, China). RAW264.7 and HaCaT cells were seeded in a 12‐well plate at a concentration of 2 × 10^5^ cells per well for overnight incubation. After treatment with various concentrations of VPNSs for 24 h, a cell live/dead assay was performed using a staining kit (Beyotime, China). The Calcein‐AM/PI working solutions were added to cells and incubated for 30 min. The cells were then washed with PBS after removing the cell culture media. The slides were finally observed under the confocal laser scanning microscope (Nikon; Tokyo, Japan). An Annexin V‐FITC/PI apoptosis detection kit (Vazyme, China) was used to analyze cell apoptosis. Briefly, after the adherent cells were blown down slightly and washed thoroughly, all cells were stained with 5 µL Annexin V‐FITC and 5 µL PI at room temperature in the dark for 10 min. The proportion of apoptotic cells was detected by flow cytometry.

### In Vitro Antibacterial Evaluation

The bacteria were diluted to a proper concentration (1 × 10^8^ CFU mL^−1^) and treated with saline and VPNSs for 2 h of incubation on a shaking incubator (200 rpm, 37 °C), respectively. Afterward, the Saline + NIR, VPNSs + NIR groups were irradiated with an 808 nm laser at a power density of 1 W cm^−2^ for 5 min. The concentration of VPNSs was 50 µg mL^−1^. For live/dead assay of *E. coli* and *S. aureus*, SYTO9 (1 µm) and PI (1 µm) were added to 50 µL of treated bacteria suspensions, and the mixtures were incubated for 15 min protected from light. Afterward, the stained bacteria were washed and imaged by the confocal laser scanning microscope (Nikon; Tokyo, Japan), with the exciting lights set at 488 and 561 nm, respectively. For bacterial morphology observation, treated samples were fixed with glutaraldehyde (2.5% w/v), dehydrated with gradient ethanol solutions, and subsequently observed using SEM and TEM (Hitachi; Tokyo, Japan) after supercritical drying and gold sputter‐coating. The bacterial suspension solution (100 µL) was taken out for Luria Borth (LB) plate cultivation at 37 °C for 24 h. The number of CFU in each plate was counted, and the viability was calculated to determine the antibacterial efficacy of the different groups.

### Ethics Statement

All surgical procedures and postoperative care of animals were performed according to the guidelines of the Institutional Animal Care and Use Committee. The animal experiment was approved by the Animal Ethical and Welfare Committee of Nanjing University (IACUC‐D2303118).

### The Treatment of Infected Wounds in vivo

Balb/C mice (6–8 weeks, female) were purchased from Shanghai Laboratory Animal Research Center (Shanghai, China). A rounded wound ≈7 mm in diameter was established on the dorsum of each mouse. Then, 10 µL of *S. aureus* solution (1 × 10^7^ CFU mL^−1^) was dropped into wounds to induce bacterial infection. After 24 h, the mice were divided randomly into four groups: PBS, PBS + NIR, VPNSs, and VPNSs + NIR. In NIR‐treated groups, the wounds were subjected to an 808 nm laser (1.0 W cm^−2^) for 10 min. A thermal imaging system (Fotric 325pro, China) recorded the temperature variations during treatment. Wound area photographs were taken on days 0, 2, 4, 6 and 8. On day 9, 3 mice of each group were sacrificed. Bacteria in infected tissue samples were quantified using standard plaque counting and the expression of macrophage markers was detected by flow cytometry. In addition, for assessment of immune responses in vivo, skins were dissected into small fragments and then digested with enzymes at 37 °C for 30 min in RPMI 1640 containing 1% P/S, 1.5 mg mL^−1^ collagenase I, 1.5 mg mL^−1^ collagenase IV, 1.5 mg mL^−1^ hyaluronidase, and 0.2 mg mL^−1^ DNase I to obtain single‐cell suspensions. The isolated cells were first blocked with anti‐CD16/CD32 to avoid non‐specific binding to the Fc receptor, followed by staining with flow cytometry antibodies as indicated. Macrophages were then stained with anti‐CD11b, anti‐F4/80, anti‐CD86, and anti‐CD206 antibodies. Flow cytometry to detect changes in macrophage polarization phenotypes. For the determination of cytokine levels in vivo, skin homogenates were centrifuged and cytokine levels of supernatant were determined using ELISA kits (MultiSciences Biotech) according to the manufacturer's instructions.

## Conflict of Interest

The authors declare no conflict of interest.

## Supporting information



Supporting Information

## Data Availability

The data that support the findings of this study are available from the corresponding author upon reasonable request.
