# Supplementary material for: Radical‐Scavenging Violet Phosphorus Nanosheets for Attenuating Hyperinflammation and Promoting Infected Wound Healing
Source: Adv Sci (Weinh). 2024 Oct 21;11(46):2407545. doi: 10.1002/advs.202407545 (PMC11633535; doi:10.1002/advs.202407545)
Supplement: Supplementary file 1 — Supporting Information [file ADVS-11-2407545-s001.pdf]

ADVANCED SCIENCE
Open Access

Supporting Information

for *Adv. Sci.*, DOI 10.1002/advs.202407545

Radical-Scavenging Violet Phosphorus Nanosheets for Attenuating Hyperinflammation and Promoting Infected Wound Healing

Zhuo Dai, Qiang Li, Meng Dang, Xiaoye Li, Ao He, Weijun Xiu, Minjin Wang, Yu Zhang, Meng Ding, Heng Dong* and Yongbin Mou**

Supporting Information

Radical-Scavenging Violet Phosphorus Nanosheets for Attenuating Hyperinflammation and Promoting Infected Wound Healing

Zhuo Dai[#], Qiang Li[#], Meng Dang[#], Xiaoye Li, Ao He, Weijun Xiu, Minjin Wang, Yu Zhang, Meng Ding, Heng Dong* and Yongbin Mou**

Z. Dai, Q. Li, M. Dang, X. Li, A. He, M. Wang, Y. Zhang, M. Ding, Prof. H. Dong and Prof. Y. Mou

Nanjing Stomatological Hospital, Affiliated Hospital of Medical School, Institute of Stomatology, Nanjing University

30 Zhongyang Road, Nanjing, Jiangsu 210008, China.

E-mail:

yongbinmou@nju.edu.cn (Y. Mou), dongheng90@smail.nju.edu.cn (H. Dong), dingmeng@smail.nju.edu.cn (M. Ding)

W. Xiu

Institute for Health Innovation and Technology

Biomedical Engineering Department

National University of Singapore

21 Lower Kent Ridge Road, Singapore 119276, Singapore

[#] These authors contributed equally to this manuscript.

Experimental Section

Materials. Nanjing Keygen Biotech Co., Ltd. (Nanjing, China) was the source of the acquisition of 4'-6-diamidino-2-phenylindole (DAPI). For all experiments, deionized water with a resistivity of 18 MΩ cm was utilized. HyClone (UT, USA) provided Dulbecco's modified Eagle's medium (DMEM), RPMI 1640 medium, penicillin sulfate and streptomycin (P/S), and phosphate-buffered saline (PBS). Fetal bovine serum (FBS) was purchased from Corning. The cell counting kit-8 (CCK-8) was acquired from Dojindo (Kumamoto, Japan). Thermo Fisher (MA, USA) provided the Pierce™ BCA protein assay kit. Calcein-AM/PI assay kit and DAPI-containing anti-fluorescence quencher were obtained from Beyotime (Shanghai, China). The mouse enzyme-linked immunosorbent assay (ELISA) kits for interleukin-6 (IL-6), necrosis factor-α (TNF-α), and interleukin-1β (IL-1β) in mice were acquired from MultiSciences. The FCM antibodies, which include anti-CD86, anti-CD206, anti-F4/80, and anti-CD11b, were bought from eBioscience (CA, USA).

Luria Broth (LB) and LB agar were obtained from Hope Biotechnology Co., Ltd. (Qingdao, China). 2,2'-azino-bis (3-ethylbenzothiazoline-6-sulphonic acid) (ABTS), superoxide dismutase (SOD) detection kit and Lipopolysaccharide (LPS) was purchased from Sigma-Aldrich (USA). Fetal bovine serum (FBS) was purchased from Gibco (CA, USA). SYTO 9/ PI Live/ Dead Bacterial Double Stain Kit, CellMask™ Plasma Membrane Stains, and CellROX Oxidative Stress Reagent were purchased from Thermo Fisher (USA). Annexin V-FITC/PI apoptosis detection kit and cell counting kit-8 (CCK-8) were purchased from Vazyme (China).

RNA-seq analysis. Total RNA from different groups was isolated using an RNA easy extraction kit (Vazyme, Nanjing, China) according to the manufacturer's recommendations, with an extra DNase treatment. For Ribosomal RNA removal, cDNA

library construction, and paired-end sequencing with NovaSeq 6000 platform (Illumina) were completed by Nanjing Personal Biotechnology Co. Ltd (China). Gene expression values were computed from fragments per kilo bases per million fragments (FPKM) values produced by the addition of a pseudocount of 1 and log2 transformation of the results.[1] Paired differential gene expression analyses were performed with DESeq2, and the criteria for identifying DEGs were as follows: expression difference multiple $|\log_2\text{Fold-Change}| > 1$ and significant *P*-value < 0.05. Volcano plots of these differential genes were drawn with ggplots2 R package. GO and KEGG enrichment analyses of differential genes were performed using topGO and clusterprofiler respectively. The STRING database was employed for protein interaction analysis to link the relationship between target genes.[2]

Protective effect of VPNSs against H_2O_2-induced cell apoptosis *in vitro*. RAW264.7 cells were seeded in 12-well culture plates at a concentration of 2×10^5 cells/well for overnight incubation. The cells were then stimulated with H_2O_2 (100 μM) or H_2O_2 + VPNSs for 12 h. An Annexin V-FITC/PI apoptosis detection kit (Vazyme, China) was used to analyze cell apoptosis as described above.

Intracellular ROS assessment. ROS generation was determined by the CellROX green reagent as a probe. RAW264.7 cells were incubated for 12 h with PBS, LPS (200 ng/mL), or LPS + VPNSs (LPS: 200 ng/mL; VPNSs: 50 μg/mL). After immediate incubation with CellROX green reagent (5 μM) for 30 min at 37 °C, cells were examined with the Sony SA3800 flow cytometer (FCM; Sony, Tokyo, Japan) or the Nikon A1 CLSM to determine ROS levels.

RT-qPCR analysis. For RT-qPCR, cDNAs were reverse transcribed with oligo-dT primers (Vazyme, Nanjing, China). The resulting cDNA was amplified and analyzed with ChamQ Universal SYBR qPCR master mix (Vazyme, Nanjing, China) by ViiA™

7 Software. Gene expression levels were normalized to those of Gapdh. The mouse primers were ordered from GeneScript (Nanjing, China). Detailed primer sequences are provided in Table S1.

Table S1. List of detailed primer sequences.

Gene	Primer (Forward)	Primer (Reverse)
iNOS	GTTCTCAGCCCAACAATACAAGA	GTGGACGGGTCGATGTCAC
Cd80	ACCCCCAACATAACTGAGTCT	TTCCAACCAAGAGAAGCGAGG
Cd86	TGTTTCCGTGGAGACGCAAG	TTGAGCCTTTGTAAATGGGCA
Il1b	GCAACTGTTCCTGAACTCAACT	ATCTTTTGGGGTCCGTCAACT
Il6	TAGTCCTTCCTACCCCAATTTCC	TTGGTCCTTAGCCACTCCTTC
Gapdh	AGGTCGGTGTGAACGGATTTG	TGTAGACCATGTAGTTGAGGTCA

Western bolt analysis. For the western bolt analysis, RAW264.7 cells were incubated overnight in 12-well plates at an initial density of 5×10^5 and treated with culture media supplemented with PBS, LPS (200 ng/mL), or LPS + VPNSs (LPS: 200 ng/mL; VPNSs: 50 μg/mL) for 12 h. Cells were then lysed with RIPA lysis buffer on ice for 30min. Protein was collected from the supernatant by centrifuging at 10,000 rpm at 4 °C for 10min in a microcentrifuge. Equal amounts of protein (20-30 μg) were reduced and denatured with 5×laemmli sample buffer, separated by SDS-PAGE gel, and transferred to the nitrocellulose membrane. After that, the membrane was blocked by 5% BSA for 1 h, incubated with the first antibody and second antibody as described by the manufacturer's instructions. Finally, the expression levels of iNOS, IL-1β, and TNF-ɑ were developed by chemiluminescence detection. The expression level of β-tubulin was utilized as the internal standard.

Photothermal effect of VPNSs. The photothermal effects of different VPNS

concentrations (25, 50, 100 and 200 μg/mL) under 808 nm NIR irradiation (1 W/cm^2, 5 min) were evaluated by using a NIR thermal imager (FLIR-E64501, FLIR Systems Inc., USA) to monitor the real-time temperature. Different laser powers (0.5, 1 and 2 W/cm^2, 5min) were then used to examine the influence on the photothermal effect. To further evaluate the photothermal stability of VPNSs, the samples were irradiated for 5 "ON/OFF" cycles under a 1 W/cm^2 laser.

Bacterial culture. *Staphylococcus aureus* (*S. aureus*, ATCC, 25923) and *Escherichia coli* (*E. coli*, ATCC, 25922) strains were used in this work. The single colony was inoculated in LB (5 mL) with shaking (220 rpm) at 37 °C overnight. The bacterial suspensions were then centrifuged at 12,000 rpm for 3 min and washed three times with saline to collect bacterial cells.

Detection of ROS generation *in vivo*. To detect ROS generated on the dorsal skin during the treatment process, ROS-sensitive dihydrofluorescein diacetate (DHFH-DA; Beyotime) was employed. The level of ROS in the skin was quantified 24 hours post-treatment as previously described, utilizing the AniView100 multi-modality live animal imaging system (BLT Photon Technology). The excitation filter operated within a wavelength range of 445 to 490 nm, while emission detection occurred between 515 and 575 nm.

Histopathological staining. Skin wound samples were dissected and fixed in 4% paraformaldehyde. Following fixation, the tissues underwent a series of processing steps: washing, dehydration, clearing, and infiltration with wax before being embedded in paraffin. Sections were then dewaxed in xylene and rehydrated through a graded alcohol series, followed by a brief rinse with distilled H_2O. For immunofluorescent analysis, sections were blocked with 3% BSA for 30 min and subsequently incubated overnight at 4 °C with primary antibodies: CD86 (Affinity, DF6332, 1:100) and iNOS

(Cell Signaling Technology, 13120, 1:200). The following day, the sections were washed with PBS, incubated with Alexa Fluor 594-conjugated donkey anti-rabbit secondary antibody at room temperature for 1 h, and mounted using a DAPI-containing mounting solution. Imaging was performed using the 3DHISTECH CaseViewer 2.4 system. For hematoxylin and eosin (H&E) staining, the sections were sequentially immersed in hematoxylin, differentiation solution, bluing agent, and eosin, with rinses in tap water between each step. Sections were then dehydrated in graded alcohols, cleared in xylene, and mounted with a xylene-based mounting medium. For Masson's trichrome staining, sections were stained consecutively with Masson A-F solutions, rinsed with 1% glacial acetic acid, dehydrated using anhydrous ethanol, cleared with xylene, and finally mounted with neutral balsam.

Statistical analysis. Data are displayed as the means ± standard deviation (SD). To determine significant differences between the two groups, the unpaired two-tailed Student's t-test was employed. Additionally, one-way or two-way ANOVA with multiple comparisons was conducted as specified. The significant differences are indicated as follows: ns, not significant, $P > 0.05$; $^{*}P < 0.05$; $^{**}P < 0.01$; $^{***}P < 0.001$ and $^{****}P < 0.0001$.

Supplemental Figures

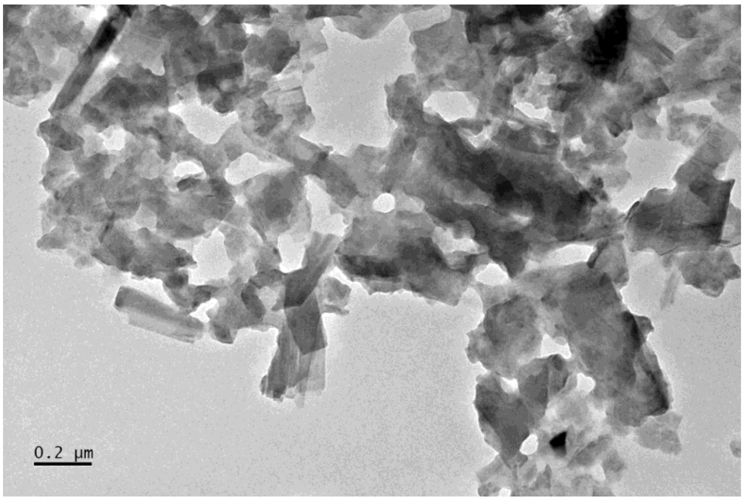

Figure S1. TEM image of VPNSs

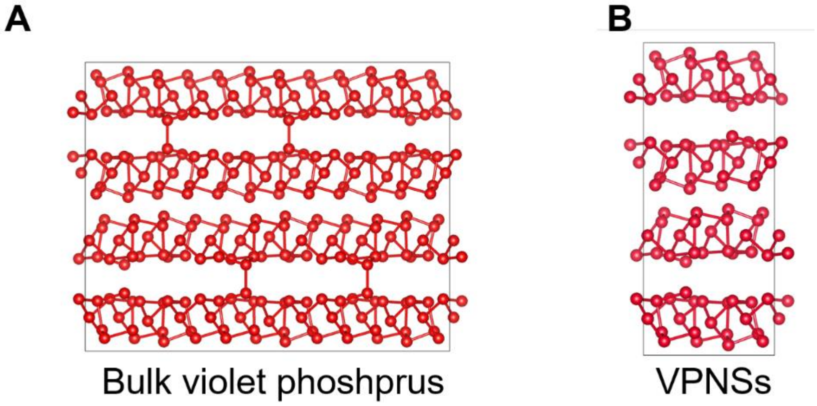

Figure S2. Crystal structural model of violet phosphorene. (A) Layered structure of violet phosphorus and (B) asymmetric unit of violet phosphorus.

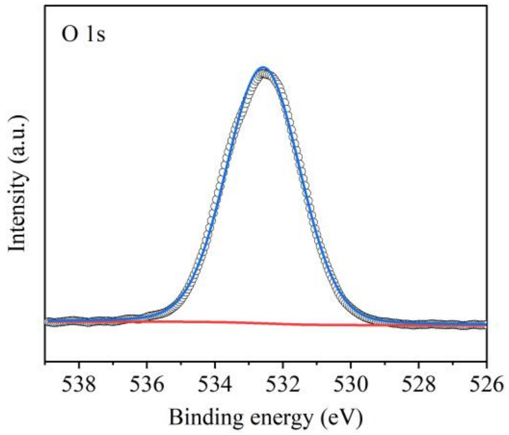

Figure S3. The XPS spectrum of O 1s for VPNSs sample.

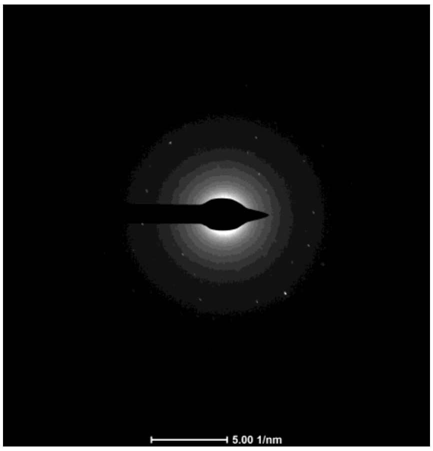

Figure S4. Electron diffraction pattern of VPNSs

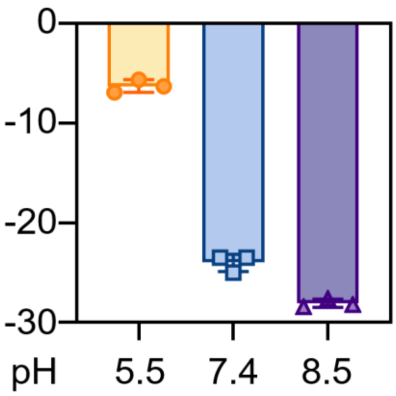

Figure S5. Zeta potentials of VPNSs at different pH conditions

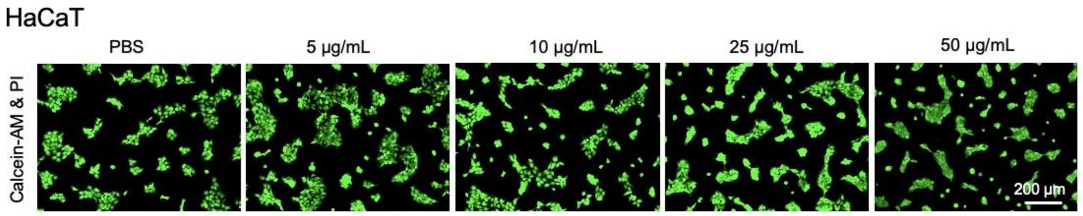

Figure S6. CLSM images of Calcein-AM/PI-stained HaCaT cells cultured with VPNSs at the indicated concentrations.

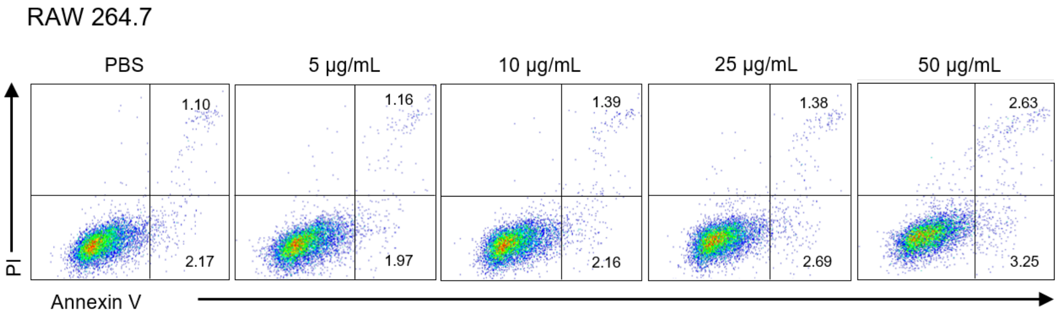

Figure S7. Cytotoxicity assay of RAW264.7 cells cultured with VPNSs at different concentrations by using the Annexin V/PI assay kit.

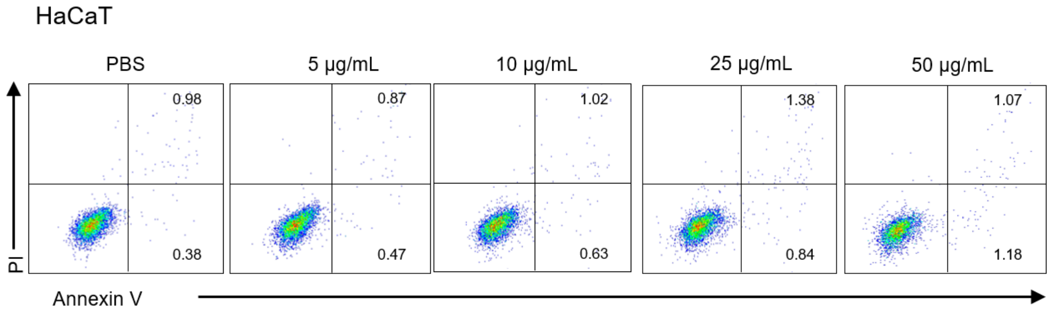

Figure S8. Cytotoxicity assay of HaCaT cells cultured with VPNSs at different concentrations by Annexin V/PI assay.

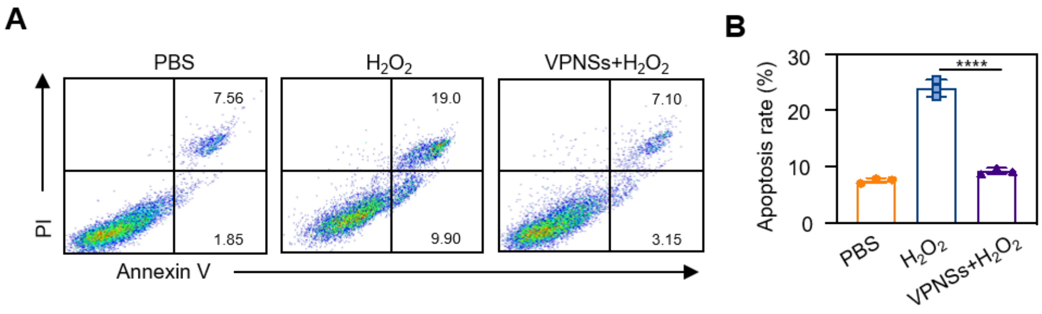

Figure S9. Cellular protection effects of VPNSs against H_2O_2-induced apoptosis. (A) Annexin V-FITC/PI flow cytometry and (B) quantitative analysis.

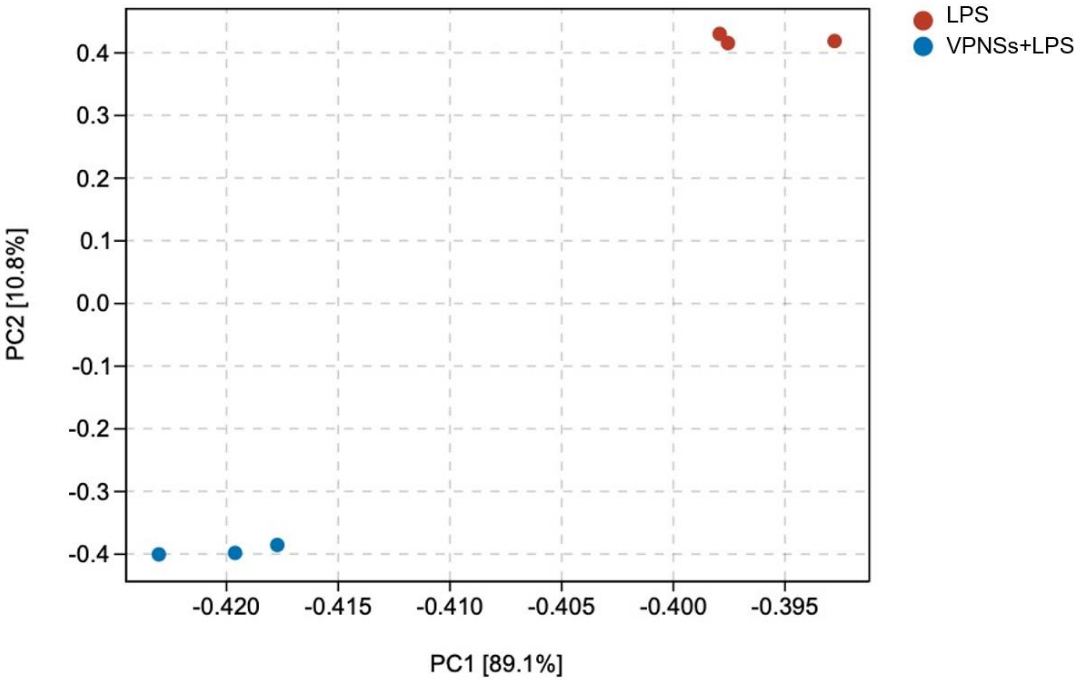

Figure S10. Principal component analysis (PCA) plot showed LPS- and VPNSs+LPS-treated RAW264.7 cells separated by their top three principal components.

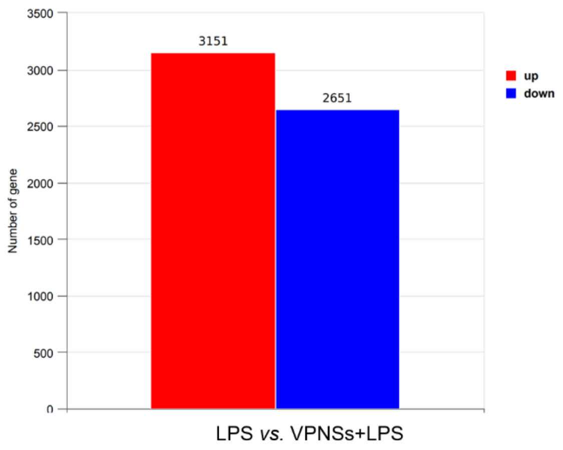

Figure S11. Differentially expressed genes (DEGs) between LPS- and VPNSs+LPS-treated RAW264.7 cells. Red, up-regulated DEGs; blue, down-regulated DEGs.

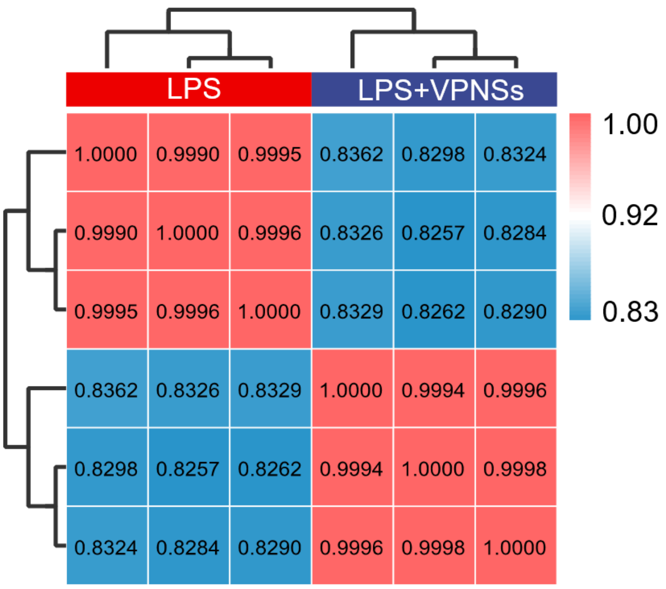

Figure S12. Heatmap showing the interactions density and clustering of samples. The color scale indicates the degree of correlation Blue, low correlation; Red, high correlation.

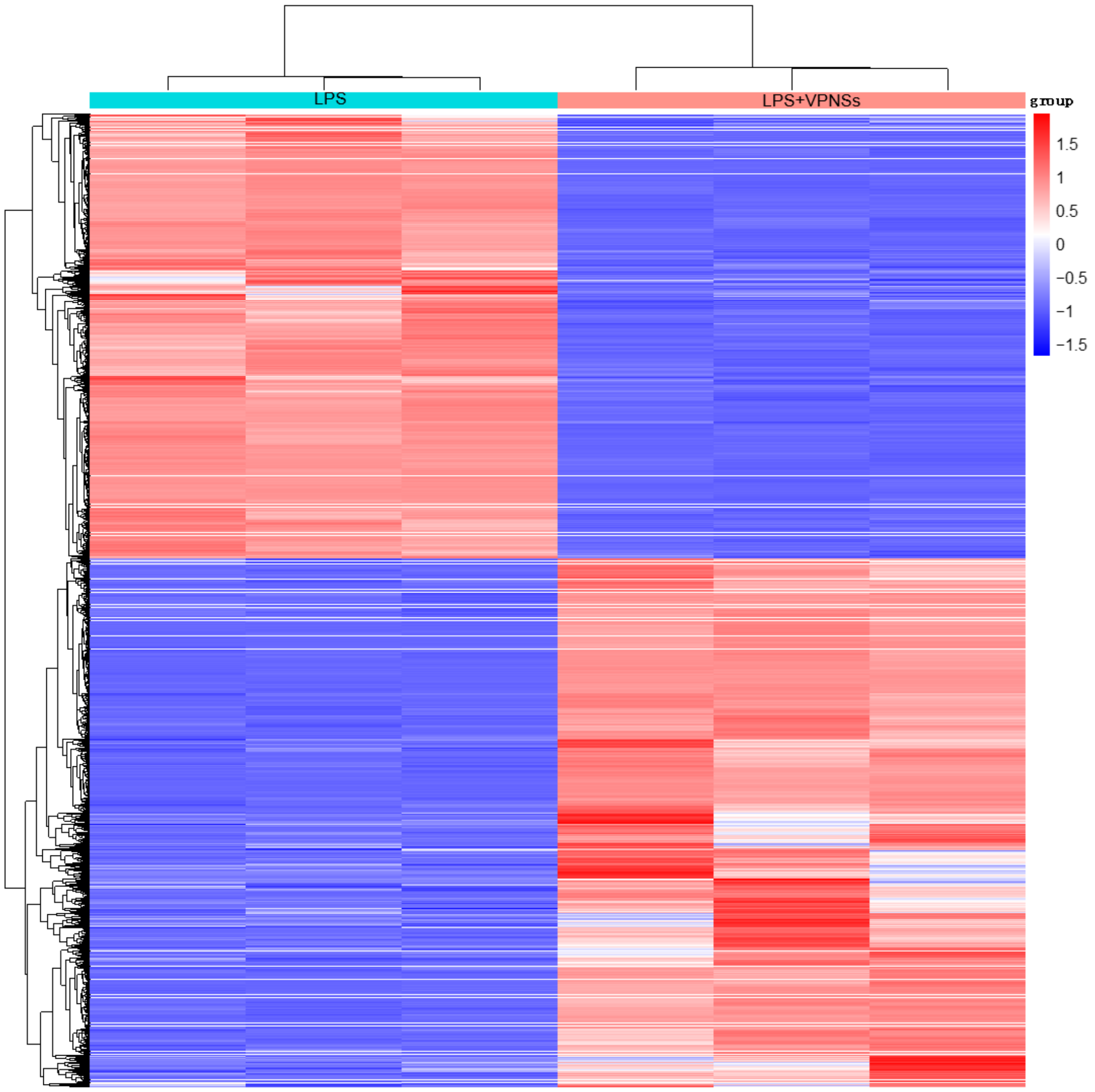

Figure S13. Heatmap of DEGs between LPS- and VPNSs+LPS- treated macrophages. The presented data comprises fragments per kilobase of exon per million fragments mapped (FPKM) values of genes in the sample, which have undergone Z-score normalization. The color scheme employed in the visualization denotes high-expression genes as red and low-expression genes as blue. Left, LPS group; right, VPNSs+LPS group.

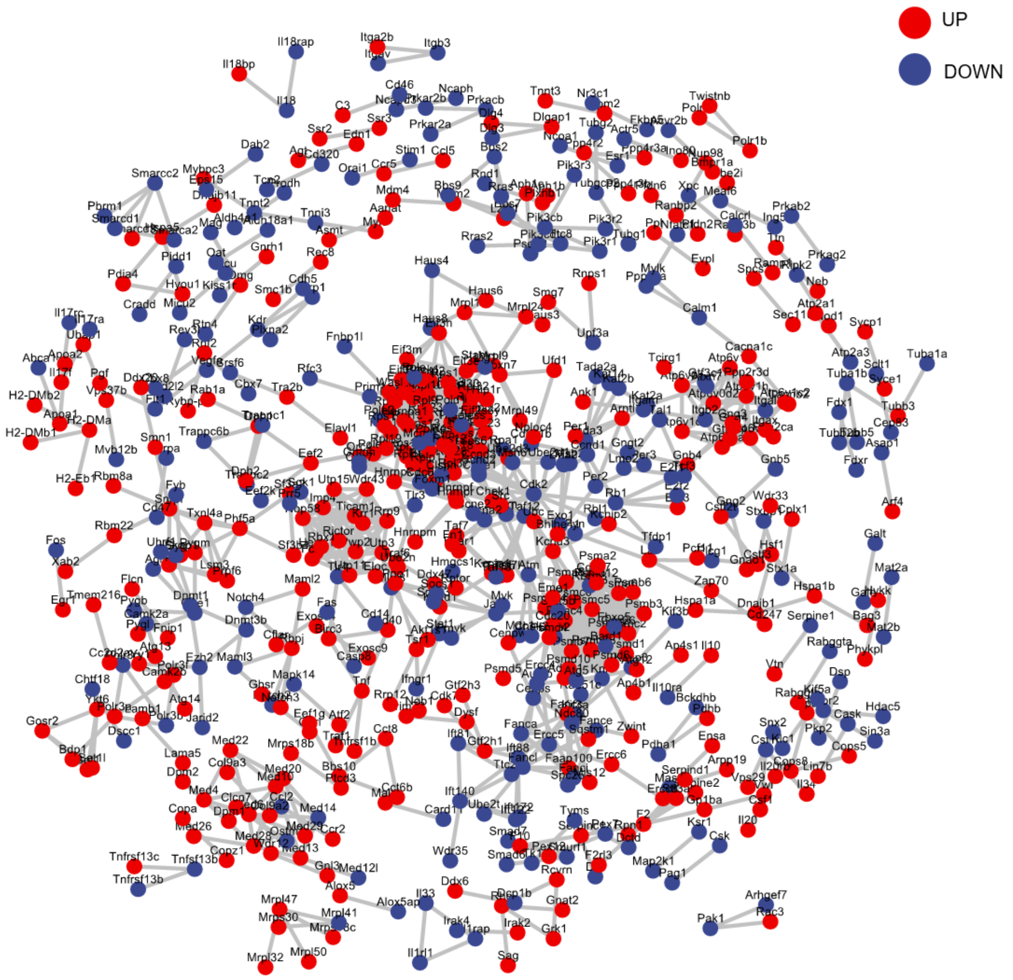

Figure S14. Protein–protein interaction (PPI) network formed by high confidence candidate genes and novel candidate genes. The network of protein interaction combined with expression level, and the points in the figure are genes (corresponding proteins), where up represents up-regulated genes and down represents down-regulated genes.

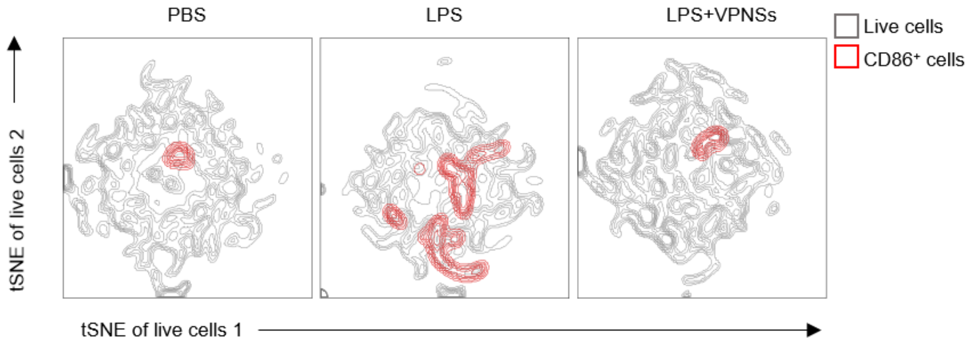

Figure S15. Flow cytometry results on CD86-positive cell groups.

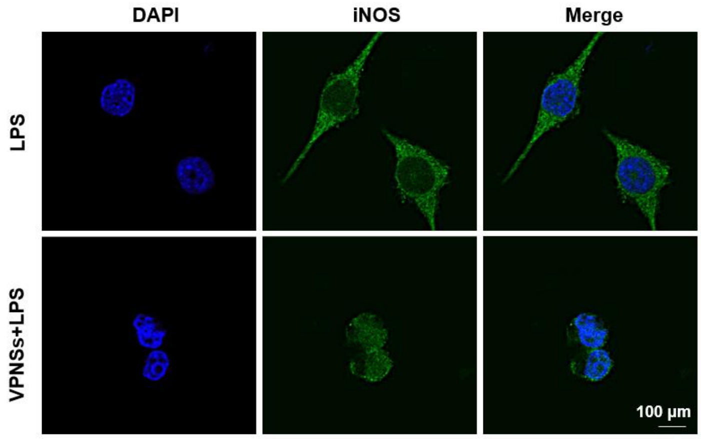

Figure S16. Confocal laser scanning microscope images of representative images of iNOS fluorescent expression of RAW264.7.

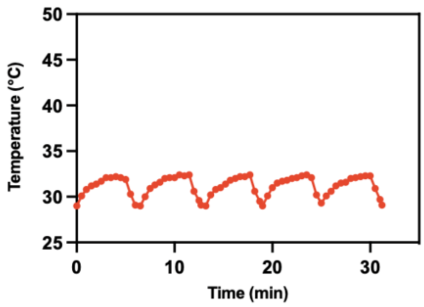

Figure S17. The photothermal stability of a PBS solution was evaluated through repeated on/off cycles of an 808 nm laser at a power density of 1.0 W/cm^2.

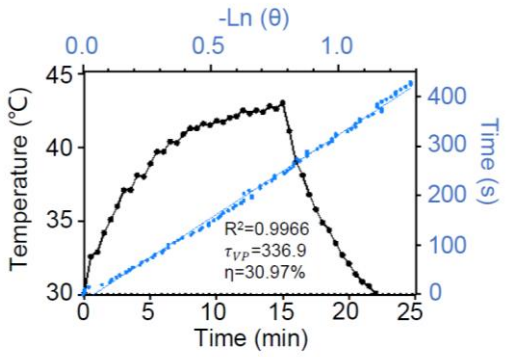

Figure S18. The photothermal conversion efficiency of VPNSs. The heat transferring time constant was determined by using the linear time data from the cooling period versus the negative natural logarithm of the driving force temperature, which was obtained from the cooling stage.

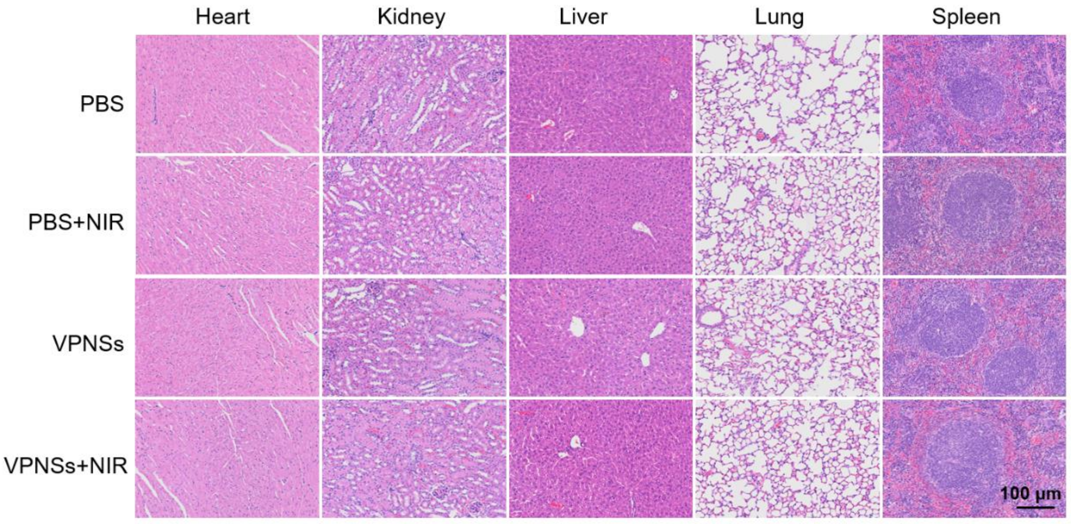

Figure S19. H&E-stained sections of major organs. The heart, liver, spleen, lung, and kidney from mice treated with either PBS or VPNSs, with and without NIR irradiation.

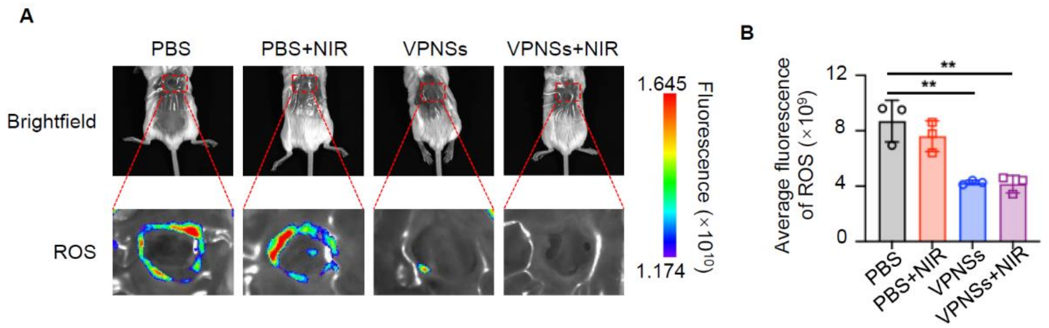

Figure S20. (A) In vivo fluorescence imaging of ROS to infected skin wounds and (B) quantification of the average fluorescence signals *in vivo* on day 3, respectively (n = 3). The statistical significance of differences between the indicated groups was analyzed using one-way ANOVA. $^{**}P < 0.01$.

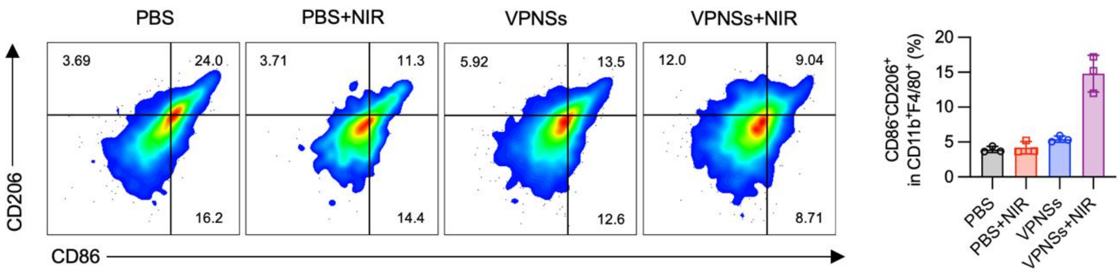

Figure S21. Flow cytometry analysis of $CD86^{-}CD206^{+}$ macrophages with different treatments on day 9.

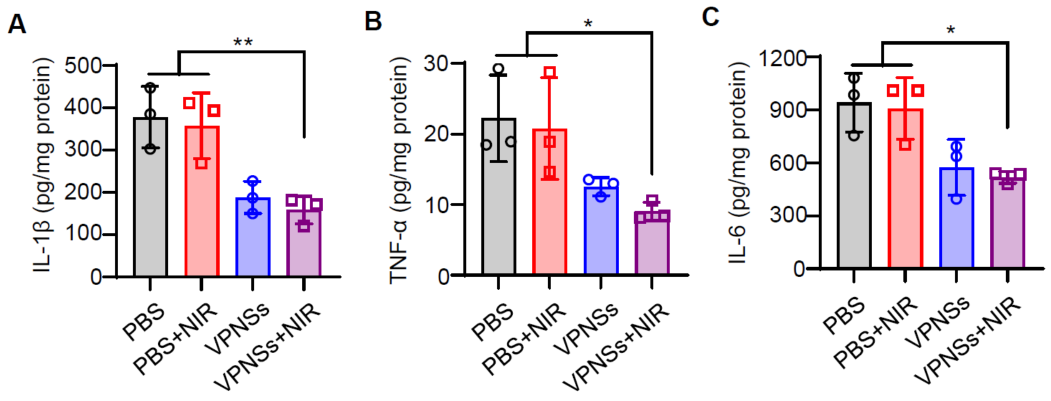

Figure S22. The release of (A) IL-1β, (B) TNF-α and (C) IL-6 in vivo after different treatments in wound tissues were measured by ELISA (n = 3). The statistical significance of differences between the indicated groups was analyzed using one-way ANOVA. *$P < 0.05$; **$P < 0.01$.

References

1. Y. Meng, L. Chen, Y. Chen, J. Shi, Z. Zhang, Y. Wang, F. Wu, X. Jiang, W. Yang, L. Zhang, C. Wang, X. Meng, Y. Wu, W. Bu, Nat Commun. 2022, 13(1), 7353.

2. D. Szklarczyk, R. Kirsch, M. Koutrouli, K. Nastou, F. Mehryary, R. Hachilif, A. L. Gable, T. Fang, N. T. Doncheva, S. Pyysalo, P. Bork, L. J. Jensen, C. von Mering, Nucleic Acids Res. 2023, 51 (D1), D638-D646.
